# The effectiveness of 0.05% cyclosporine A combined with deproteinized calf blood extract eye gel in the treatment of dry eye disease after pterygium surgery

**DOI:** 10.3389/fmed.2026.1838660

**Published:** 2026-07-14

**Authors:** Yingxin Chen, Danni Wang, Qingqing Zhao, Ruiyao Gao, Zhida You

**Affiliations:** Department of Ophthalmology, General Hospital of Northern Theater Command, Shenyang, China

**Keywords:** cyclosporine A, deproteinized calf blood extract, dry eye disease, pterygium surgery, tear film stability

## Abstract

**Purpose:**

To investigate the effectiveness of combining 0.05% cyclosporine A (CsA) eye drops and deproteinized calf blood extract (DCBE) eye gel for treating dry eye disease (DED) following pterygium surgery.

**Methods:**

This retrospective study included 64 patients (32 in each group) who underwent pterygium excision with autologous limbal stem cell transplantation. The experimental group received 0.05% CsA eye drops and DCBE eye gel, while the control group received DCBE eye gel alone. Ocular parameters were assessed preoperatively and at 1, 2, and 3 months postoperatively.

**Results:**

Repeated-measures ANOVA demonstrated significant interaction effects between time and group for tear fluorescein break-up time (TFBUT; *p* < 0.001), ocular surface disease index (OSDI; *p* = 0.005), and Schirmer *I* test (SIT; *p* < 0.001). The experimental group had higher TFBUT, lower OSDI, and better SIT outcomes than the control group at all postoperative time points (*p* < 0.05). Corneal fluorescein staining (CFS) scores were also lower in the experimental group compared to the control group at 1, 2, and 3 months (*p* < 0.05). During the 3-month follow-up, the incidence of complications was 9.38% in the control group and 6.25% in the experimental group (*p* = 0.641), with no recurrence of pterygium observed in either group.

**Conclusion:**

The combination of 0.05% CsA eye drops and DCBE eye gel is more effective than DCBE eye gel alone in improving tear film stability and treating DED following pterygium surgery, with good safety and no recurrence observed.

## 1 Introduction

Pterygium is a prevalent ocular surface disorder characterized by the encroachment of degenerative fibrovascular tissue from the bulbar conjunctiva toward the cornea (1). Although its etiology remains incompletely understood, pterygium is known to have significant clinical implications (2). It not only affects cosmetic appearance but also extends toward the central cornea, potentially causing severe visual impairment and ocular discomfort, such as dryness, foreign body sensation, and photophobia, when it encroaches upon the pupillary area (3). Accumulating evidence suggests a close association between pterygium and dry eye disease (DED) (4–6). DED is an ocular condition resulting from abnormal tear composition, insufficient tear secretion, or tear circulation disorders, with the core pathological mechanism being the disruption of tear film homeostasis (7). This disruption manifests as ocular discomfort and ocular surface tissue damage, including blurred vision, dryness, foreign body sensation, photophobia, and tearing, thereby significantly impacting patients’ quality of life (8). In patients with pterygium, DED is often exacerbated due to conjunctival metaplasia, tear film instability, and meibomian gland dysfunction, which induce local changes in ocular surface homeostasis (9). These changes are characterized by inflammation, disruption of Bowman’s layer, epithelial metaplasia, active fibrovascular tissue, aggregation of limbal stromal cells, and a reduction in goblet cell density (10).

In clinical practice, surgical intervention is the primary treatment modality for pterygium, and the selection of appropriate surgical techniques is crucial for minimizing recurrence rates (11). Common surgical procedures include pterygium excision combined with autologous limbal stem cell transplantation, conjunctival autograft transplantation, or amniotic membrane transplantation, all of which have demonstrated relatively low recurrence rates (12, 13). Among these, pterygium excision combined with autologous limbal stem cell transplantation is particularly effective in improving vision and restoring ocular surface integrity by utilizing well-matched, autologous tissue (14). However, the procedure may disrupt tear film function, leading to postoperative DED (14). Severe postoperative DED can even contribute to pterygium recurrence (15). Given the high incidence of postoperative DED, perioperative management of ocular surface health has become a priority. In recent years, local pharmacological agents have been increasingly utilized in the perioperative period to mitigate postoperative DED (16, 17). Deproteinized calf blood extract (DCBE) eye gel has gained widespread application for postoperative corneal and conjunctival tissue repair and symptom improvement (18). It forms a protective film on the corneal surface, reduces suture friction, protects and lubricates the epithelium, and alleviates foreign body sensation (18). Additionally, it contains various nutrients that promote corneal epithelial cell metabolism and regeneration, thereby enhancing tear film stability (19). Nevertheless, monotherapy is often insufficient, and combination therapy is commonly employed in clinical practice (19).

0.05% cyclosporine A (CsA) eye drops have demonstrated efficacy in treating DED by inhibiting the calcineurin-phosphatase pathway, leading to increased natural tear production and goblet cell density (20). Furthermore, it reduces the expression of conjunctival inflammatory markers, exerts anti-inflammatory effects, increases the expression of transforming growth factor-β2, and plays an immunomodulatory role (21, 22). It can also enhance the density and sensitivity of sub-basal corneal nerves, thereby improving visual function and maintaining ocular surface health (23, 24). Although numerous studies have investigated the use of CsA for preventing pterygium recurrence (25–27), its therapeutic efficacy in postoperative dry eye following pterygium surgery remains inconclusive and warrants further investigation. A study has demonstrated that long-term topical application of CsA after pterygium excision can improve tear secretion (28). Another study also reported that topical CsA significantly increased tear fluorescein break-up time (TFBUT) and Schirmer I (SIT) values after pterygium surgery (29). However, the impact mechanism of combining 0.05% CsA eye drops with DCBE eye gel on postoperative tear film stability has not been systematically elucidated. This study aims to address this research gap by investigating the clinical efficacy of combining 0.05% CsA eye drops with DCBE eye gel in patients with postoperative dry eye following pterygium surgery, thereby providing a novel intervention strategy for the management of postoperative dry eye.

## 2 Materials and methods

### 2.1 Study design

This retrospective study included patients who underwent pterygium excision combined with autologous limbal stem cell transplantation at the Department of Ophthalmology, General Hospital of Northern Theater Command, between August 2023 and August 2024. Patients were assigned to either the experimental group or the control group based on their postoperative treatment regimen for DED. The experimental group received a combination of 0.05% CsA eye drops and DCBE eye gel (both from Sinqi Pharmaceutical, Shenyang, China), while the control group received only the DCBE eye gel. This study was approved by the Ethics Committee of the General Hospital of Northern Theater Command of the People’s Liberation Army (Approval No. Y [2024] 343), and informed consent was obtained from all participants.

### 2.2 Inclusion and exclusion criteria

Patients were included if they had undergone first-time pterygium excision with autologous limbal stem cell transplantation and met at least one of the following postoperative dry eye diagnostic criteria (30): (1) 5 s < TFBUT ≤ 10 s or 5 mm/5 min < SIT (without anesthesia) ≤ 10 mm/5 min; (2) at least one subjective symptom (e.g., dryness, foreign body sensation, burning, fatigue, or visual fluctuation) assessed by binary response (yes/no) based on patient interview; and (3) positive corneal/conjunctival fluorescein staining defined as a score ≥1 on a 0–3 grading scale. Alternatively, patients with TFBUT ≤ 5 s or SIT ≤ 5 mm/5 min were included if they experienced any of the aforementioned subjective symptoms. Additionally, patients had to receive initial treatment for postoperative dry eye, comply with ophthalmic examinations, and have a follow-up duration of ≥3 months.

Patients were excluded if they met any of the following criteria: (1) Use of corticosteroids, artificial tears, or nonsteroidal anti-inflammatory drugs within 1 month preoperatively. (2) History of ocular trauma or previous ocular surgery. (3) Presence of congenital alacrima, glaucoma, ocular hypertension, iritis, acute ocular surface infections, inflammatory conditions, grade 3 ocular hyperemia, or any other ocular pathology unrelated to pterygium. (4) Systemic diseases (e.g., connective tissue diseases, metabolic disorders) that could contribute to dry eye. (5) Allergy to CsA eye drops or DCBE eye gel. (6) Age < 18 years, presence of psychiatric/psychological disorders, or poor compliance. (7) Severe hepatic or renal dysfunction, cardiovascular or hematologic disorders. (8) Pregnancy or lactation. (9) Participation in other clinical trials during the follow-up period.

### 2.3 Surgical procedure and postoperative medication

Preoperatively, comprehensive medical history reviews and ophthalmic examinations, including visual acuity, refraction, intraocular pressure measurement, slit-lamp examination, and anterior segment photography, were conducted to exclude surgical contraindications. The Tan classification was employed for grading pterygium based on its morphological and size characteristics observed under a slit-lamp microscope (Topcon SL-D701, at 10× or 16× magnification) (31).

All surgeries were performed by a single surgeon under identical microscope conditions with topical anesthesia. A 1 mL injection of 2% lidocaine was administered via temporal subconjunctival infiltration. After inserting a lid speculum and irrigating the conjunctival sac with sterile saline, a conjunctival incision was made at the semilunar fold. The pterygium was dissected from the head and neck to the limbus while preserving the lateral rectus muscle. The defect dimensions were measured, and a limbal-conjunctival graft, 1–2 mm larger than the defect, was harvested from the superotemporal quadrant and sutured to the sclera with 10–0 nylon interrupted sutures. Postoperatively, a silicone hydrogel bandage contact lens was placed, tobramycin/dexamethasone ointment was applied, and pressure patching was performed for 24 h. Sutures were removed 10–14 days later. At that time, a slit-lamp examination was routinely performed to assess corneal epithelial integrity and exclude obvious signs of infection or inflammation.

Pre- and postoperative medication regimens were standardized. For infection prophylaxis, all patients received levofloxacin (one drop, four times daily for 3 days) preoperatively. Postoperatively, the experimental group received 0.05% CsA eye drops (one drop, twice daily) and DCBE eye gel (one drop, four times daily) for 3 months, while the control group received DCBE eye gel alone. Standardized postoperative therapy included levofloxacin (four times daily), prednisolone acetate (tapered from four to one dose/day over 4 weeks), and tobramycin/dexamethasone ointment (0.1 mg nightly for 2 weeks). All topical medications were administered with intervals of at least 5 min.

### 2.4 Assessments of ocular parameters

Postoperative assessments were performed by a single ophthalmologist at 1, 2, and 3 months. The assessments included a variety of measures to evaluate ocular surface status and potential complications. Specifically, the ocular surface disease index (OSDI) questionnaire, based on 12 questions about ocular surface symptoms, was used to evaluate the severity of DED. Scores range from 0 to 100, with higher scores indicating more severe symptoms. Additionally, tear fluorescein break-up time (TFBUT) and corneal fluorescein staining (CFS) scoring were conducted using a fluorescein sodium ophthalmic strip (Tianjin Jingming New Technological Development Co., Ltd., Tianjin, China) moistened with one drop of chloramphenicol eye drop, placed in the medial one-third of the subject’s lower conjunctival sac. CFS was graded on a 0–3 scale: 0, no staining; 1, scattered punctate staining; 2, numerous punctate staining without confluent patches; 3, confluent patches of staining. It should be noted that this CFS scoring system specifically quantifies fluorescein uptake by damaged corneal epithelium, which is distinct from standard grading systems such as the Efron grading scale that assess conjunctival hyperemia. The SIT was also performed to assess basal tear secretion by measuring the length of the wetted portion of a Schirmer strip placed in the medial one-third of the patient’s lower conjunctival sac after 5 min. Furthermore, tear meniscus height (TMH) was measured non-invasively using the ocular surface analyzer (MediWorks Precision Instruments Co., Ltd., Shanghai, China), with a normal value > 0.2 mm and values ≤ 0.2 mm suggesting aqueous-deficient dry eye. Lastly, ocular redness was graded by a single ophthalmologist using a slit-lamp microscope, with grades 0–2 considered normal and grade 3 indicating an abnormal finding and an absolute contraindication for surgery, suggesting active ocular infection or acute inflammatory response. Throughout the follow-up period, complications were closely monitored.

### 2.5 Statistical analysis

Statistical analysis was performed using SPSS version 28.0. Quantitative data were expressed as mean ± standard deviation. Repeated-measures analysis of variance (ANOVA) was used to assess the effects of group and time on various indicators, with the Greenhouse–Geisser correction applied when the assumption of sphericity was not met. Simple effect analysis was conducted when there was an interaction between group and time; otherwise, main effect analysis was performed. *Post hoc* comparisons were made using the LSD-*t*-test. For independent samples, *t*-tests were used for continuous variables between the two groups (age, TFBUT, OSDI, SIT, CFS, TMH), while categorical variables were analyzed using the *χ*^2^ test (gender, eye side) or Fisher’s exact test (dry eye grade, pterygium grade). The Wilcoxon rank-sum test was applied for ordinal data (ocular redness grade). A *p*-value of less than 0.05 was considered statistically significant.

## 3 Results

### 3.1 Baseline characteristics

**Figure 1:**
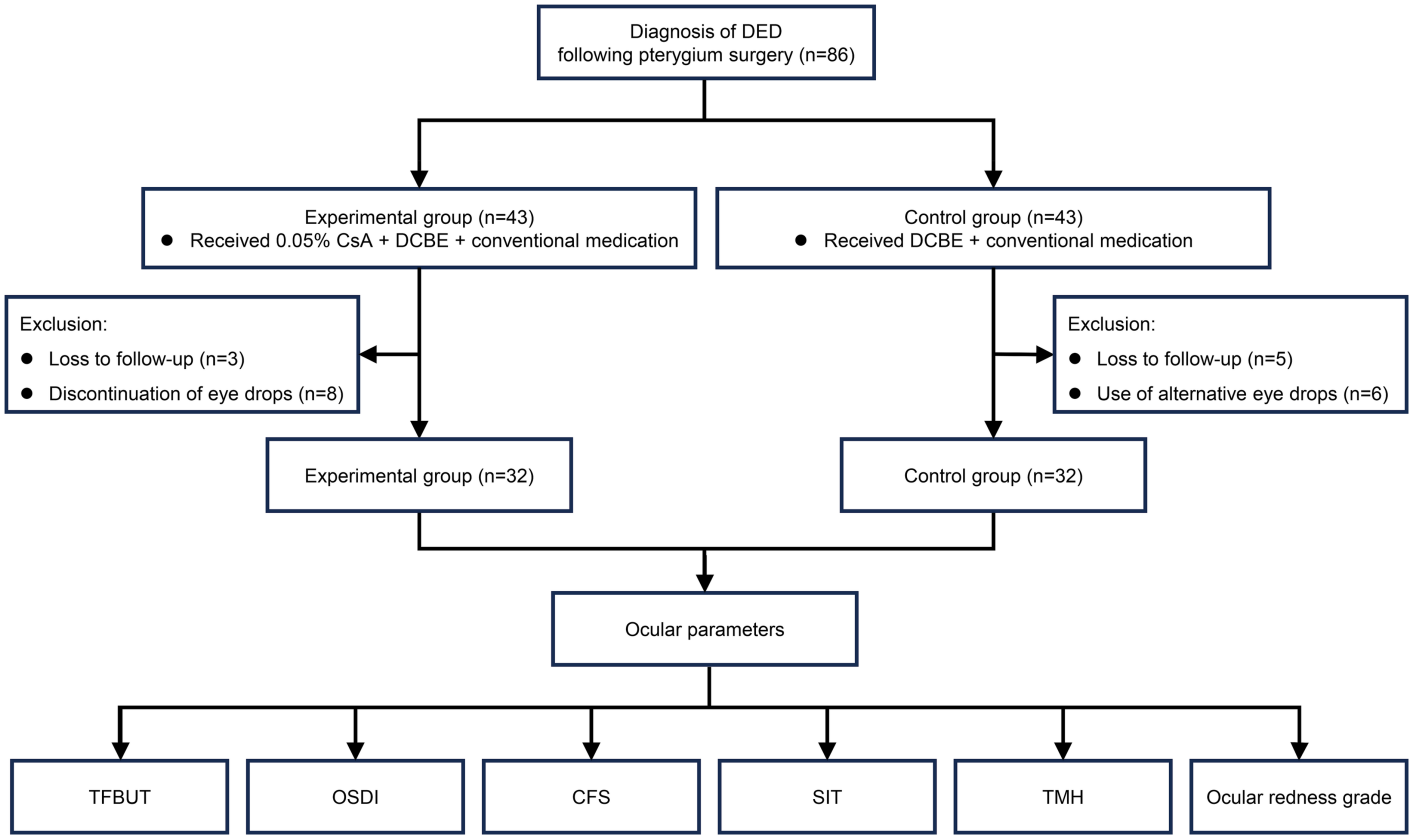
The flow diagram in the study. DED, dry eye disease; CsA, cyclosporine A; DCBE, deproteinized calf blood extract; TFBUT, tear fluorescein break-up time; OSDI, ocular surface disease index; CFS, corneal fluorescein staining; SIT, Schirmer *I* test; TMH, tear meniscus height.

This study included 86 eyes of 86 patients who developed dry eye following pterygium excision between August 2023 and August 2024. During the follow-up period, eight patients were lost to follow-up (five in the control group and three in the experimental group). Additionally, eight patients in the experimental group were excluded for discontinuing the use of eye drops, and six patients in the control group were excluded for using alternative eye drops postoperatively. Ultimately, 64 eyes of 64 patients (32 eyes in the control group and 32 eyes in the experimental group) completed the 3-month follow-up. The flow diagram is illustrated in Figure 1. Preoperatively, there were no significant differences between the two groups in terms of pterygium grade, dry eye grade, or related ocular parameters (*p* > 0.05 for all comparisons, Table 1), indicating clinical comparability.

**Table 1:** Comparison of baseline characteristics between control and experimental groups.

Variable	Control group (*n* = 32)	Experimental group (*n* = 32)	*p*-value
Gender (*n*, %)			0.451
Male	16 (50.00)	13 (40.63)	
Female	16 (50.00)	19 (59.37)	
Eye side (*n*, %)			0.308
Right	11 (34.38)	15 (46.88)	
Left	21 (65.62)	17 (53.12)	
Age (years, mean ± SD)	63.97 ± 5.27	65.62 ± 6.61	0.274
Pterygium grade (*n*, %)			0.731
Grade 1	3 (9.38)	5 (15.63)	
Grade 2	20 (62.50)	18 (56.25)	
Grade 3	9 (28.12)	9 (28.12)	
Dry eye grade (*n*, %)			1.000
Mild	27 (84.37)	27 (84.37)	
Moderate	5 (15.63)	5 (15.63)	
Preoperative TFBUT (s, mean ± SD)	7.20 ± 1.94	7.14 ± 2.03	0.911
Preoperative OSDI (mean ± SD)	19.22 ± 4.28	19.14 ± 4.23	0.940
Preoperative CFS (mean ± SD)	1.90 ± 0.55	1.88 ± 0.59	0.836
Preoperative SIT (mm/5 min, mean ± SD)	5.90 ± 1.14	5.82 ± 1.11	0.751
Preoperative TMH (mm, mean ± SD)	0.16 ± 0.03	0.17 ± 0.05	0.639
Preoperative ocular redness grade (*n*, %)			0.796
Grade 1	11 (34.38)	12 (37.50)	
Grade 2	21 (65.62)	20 (62.50)	

TFBUT, tear fluorescein break-up time; OSDI, ocular surface disease index; CFS, corneal fluorescein staining; SIT, Schirmer *I* test; TMH, tear meniscus height.

### 3.2 Ocular parameters

#### 3.2.1 TFBUT

**Figure 2:**
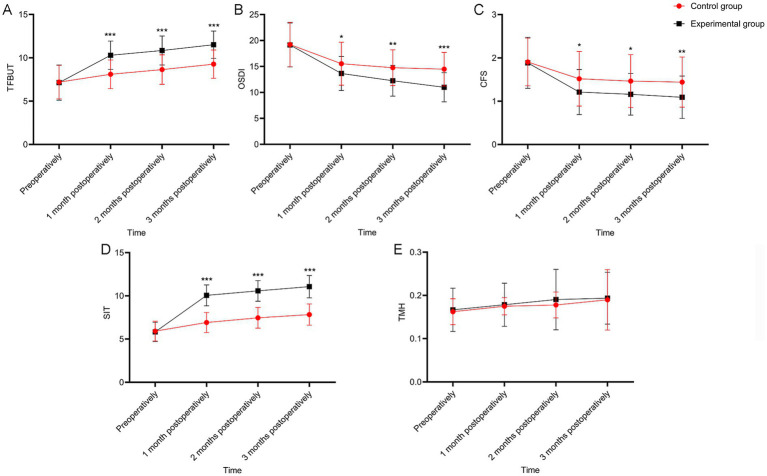
Dynamic changes in ocular parameters for experimental and control groups over time. **(A)** Tear fluorescein break-up time (TFBUT). **(B)** Ocular surface disease index (OSDI). **(C)** Corneal fluorescein staining (CFS). **(D)** Schirmer *I* test (SIT). **(E)** Tear meniscus height (TMH). ^*^Significantly different from the control group (^*^p < 0.05, ^**^p < 0.01, ^***^p < 0.001).

Repeated-measures ANOVA demonstrated a significant interaction effect between time and group for TFBUT (*p* < 0.001). Preoperatively, the TFBUT values were comparable between the control and experimental groups (*p* = 0.911, Table 2 and Figure 2A). However, postoperatively, the experimental group exhibited significantly higher TFBUT values at 1, 2, and 3 months compared to the control group (*p* < 0.001 at each time point, Table 2 and Figure 2A). This improvement was consistent across all postoperative time points, with TFBUT values increasing progressively from 1 to 3 months in both groups (*p* < 0.05 for all comparisons, Table 2 and Figure 2A).

**Table 2:** Comparison of ocular parameters between control and experimental groups.

Variable	Control group (*n* = 32)	Experimental group (*n* = 32)	*p*-value^*^
TFBUT (s, mean ± SD)			
Preoperatively	7.20 ± 1.94	7.14 ± 2.03	0.911
1 month postoperatively	8.10 ± 1.65^a^	10.30 ± 1.63^*,a^	<0.001
2 months postoperatively	8.64 ± 1.71^a,b^	10.85 ± 1.67^*,a,b^	<0.001
3 months postoperatively	9.28 ± 1.64^a,b,c^	11.52 ± 1.58^*,a,b,c^	<0.001
OSDI (mean ± SD)			
Preoperatively	19.22 ± 4.28	19.14 ± 4.23	0.940
1 month postoperatively	15.53 ± 4.14^a^	13.65 ± 3.28^*,a^	0.048
2 months postoperatively	14.77 ± 3.45^a^	12.25 ± 2.97^*,a,b^	0.003
3 months postoperatively	14.49 ± 3.23^a^	10.99 ± 2.80^*,a,b,c^	<0.001
CFS (mean ± SD)			
Preoperatively	1.90 ± 0.55	1.88 ± 0.59	0.836
1 month postoperatively	1.52 ± 0.63^a^	1.21 ± 0.52^*,a^	0.029
2 months postoperatively	1.47 ± 0.61^a^	1.16 ± 0.48^*,a^	0.023
3 months postoperatively	1.44 ± 0.58^a^	1.09 ± 0.49^*,a^	0.010
SIT (mm/5 min, mean ± SD)			
Preoperatively	5.90 ± 1.14	5.82 ± 1.11	0.751
1 month postoperatively	6.91 ± 1.17^a^	10.05 ± 1.21^*,a^	<0.001
2 months postoperatively	7.46 ± 1.20^a,b^	10.57 ± 1.20^*,a,b^	<0.001
3 months postoperatively	7.82 ± 1.23^a,b,c^	11.06 ± 1.30^*,a,b,c^	<0.001
TMH (mm, mean ± SD)			
Preoperatively	0.16 ± 0.03	0.17 ± 0.05	0.639
1 month postoperatively	0.17 ± 0.02	0.18 ± 0.05	0.685
2 months postoperatively	0.18 ± 0.03	0.19 ± 0.07	0.395
3 months postoperatively	0.19 ± 0.07	0.19 ± 0.06	0.827
Ocular redness grade (*n*, %)			
Preoperatively			0.796
Grade 1	11 (34.38)	12 (37.50)	
Grade 2	21 (65.62)	20 (62.50)	
1 month postoperatively			0.487
Grade 1	15 (46.88)^a^	17 (53.12)^a^	
Grade 2	14 (43.75)	14 (43.75)	
Grade 3	3 (9.38)	1 (3.12)	
2 months postoperatively			0.536
Grade 1	22 (68.75)^a,b^	24 (75.00)^a,b^	
Grade 2	9 (28.12)	8 (25.00)	
Grade 3	1 (3.12)	0 (0.00)	
3 months postoperatively			0.270
Grade 1	26 (81.25)^a,b,c^	29 (90.62)^a,b,c^	
Grade 2	5 (15.62)	3 (9.38)	
Grade 3	1 (3.12)	0 (0.00)	

^a^Significantly different from preoperative values (*p* < 0.05); ^b^Significantly different from 1-month postoperative values (*p* < 0.05); ^c^Significantly different from 2-month postoperative values (*p* < 0.05); ^*^Significantly different from the control group (*p* < 0.05).

TFBUT, tear fluorescein break-up time; OSDI, ocular surface disease index; CFS, corneal fluorescein staining; SIT, Schirmer *I* test; TMH, tear meniscus height.

#### 3.2.2 OSDI

Similar to TFBUT, a significant interaction effect between time and group was observed for OSDI (*p* = 0.005). Preoperatively, no significant difference in OSDI was noted between the two groups (*p* = 0.940, Table 2 and Figure 2B). Postoperatively, the experimental group consistently had lower OSDI scores than the control group at 1, 2, and 3 months (*p* < 0.05 at each time point, Table 2 and Figure 2B). In the experimental group, OSDI scores decreased significantly from preoperative levels and continued to decline progressively over the 3-month follow-up period (*p* < 0.05 for all comparisons, Table 2 and Figure 2B). In contrast, although the control group also showed a significant reduction in OSDI from preoperative levels (*p* < 0.05, Table 2 and Figure 2B), no significant differences were observed between the postoperative time points themselves (*p* > 0.05, Table 2 and Figure 2B).

#### 3.2.3 CFS

**Figure 3:**
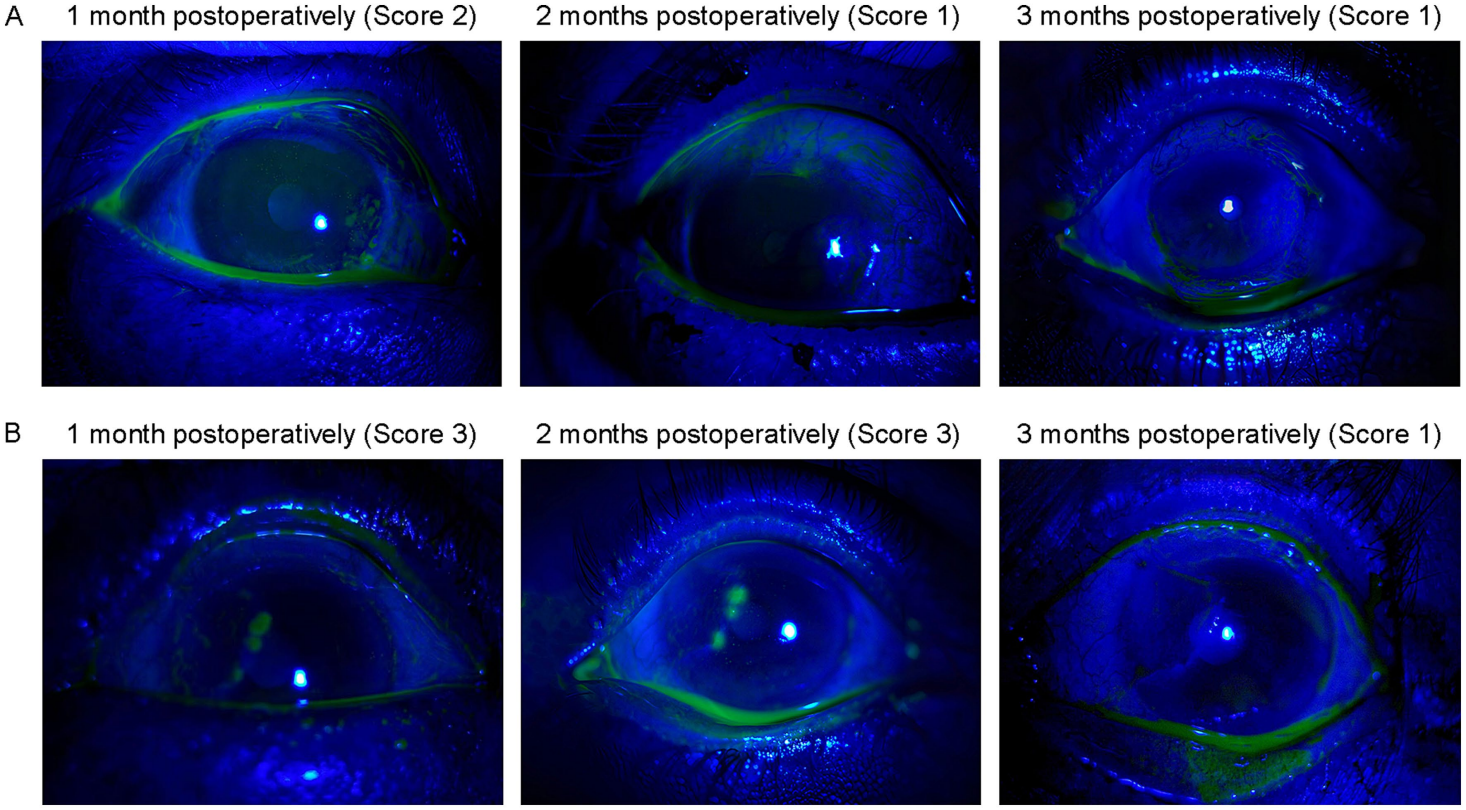
Anterior segment photographs of corneal fluorescein staining (CFS). **(A)** A patient from the experimental group at 1, 2, and 3 months postoperatively. **(B)** A patient from the control group at 1, 2, and 3 months postoperatively.

The analysis of CFS scores showed significant main effects of time (*p* < 0.001) and group (*p* = 0.001), but no significant interaction effect between time and group (*p* = 0.283). Preoperatively, CFS scores were comparable between the two groups (*p* = 0.836, Table 2 and Figure 2C). Postoperatively, the experimental group had significantly lower CFS scores compared to the control group at 1, 2, and 3 months (*p* < 0.05 at each time point, Table 2 and Figures 2C, 3). Overall, CFS scores in both groups were significantly lower at all postoperative time points compared to preoperative values. However, within-group comparisons at each postoperative time point did not reveal significant differences (*p* > 0.05, Table 2 and Figure 2C).

#### 3.2.4 SIT

The SIT results also demonstrated a significant interaction effect between time and group (*p* < 0.001). Preoperatively, no significant difference in SIT measurements was observed between the two groups (*p* = 0.751, Table 2 and Figure 2D). Postoperatively, the experimental group exhibited significantly better SIT outcomes compared to the control group at 1, 2, and 3 months (*p* < 0.05 at each time point, Table 2 and Figure 2D). Both groups showed significant improvements in SIT values at each postoperative time point compared to preoperative levels, with a clear temporal gradient effect: SIT values increased progressively from 1 to 3 months (*p* < 0.05 for all comparisons, Table 2 and Figure 2D).

#### 3.2.5 TMH

In contrast to the aforementioned parameters, repeated-measures ANOVA for TMH showed no significant interaction between time and group (*p* = 0.639). Therefore, the analysis focused on the main effects. The main effect of group revealed no significant difference in TMH between the two groups (*p* = 0.391). However, the main effect of time indicated an overall upward trend in TMH over time (*p* = 0.019, Figure 2E), although within-group comparisons of TMH at each time point before and after surgery did not reach statistical significance (*p* > 0.05, Figure 2E).

#### 3.2.6 Ocular redness grading

Preoperatively, no significant difference in the grading of ocular redness was observed between the two groups (*p* = 0.796). At each postoperative time point, comparisons of ocular redness grading between the two groups also showed no significant differences (*p* > 0.05, Table 2). However, in both groups, the grading of ocular redness was significantly lower at all postoperative follow-up time points compared to preoperative levels, with a clear temporal trend: the grading decreased progressively from 1 to 3 months (*p* < 0.05 for all within-group comparisons, Table 2).

### 3.3 Complications

During the 3-month postoperative follow-up period, the control group experienced three complications, including one case of conjunctival congestion, one case of blurred vision, and one case of eye itching. The overall incidence rate of complications in the control group was 9.38% (3/32). In the experimental group, two complications were observed, including one case of eye itching and one case of blurred vision, with an overall incidence rate of 6.25% (2/32). There was no significant difference in the incidence of complications between the two groups (*p* = 0.641). Importantly, during the 3-month follow-up period, no recurrence of pterygium was observed in either group.

## 4 Discussion

DED is a common and distressing complication following pterygium surgery, significantly impairing patients’ postoperative quality of life and disease prognosis (32). The development of DED after pterygium surgery is multifactorial, involving the disruption of tear film dynamics, mechanical injury-induced inflammatory response, and corneal nerve damage (33). These factors interact synergistically, destabilizing the tear film and leading to the occurrence or exacerbation of DED, and may even induce pterygium recurrence (15). Notably, pterygium morphology, including size, shape, redness and fleshy appearance, correlates with disease activity and severity (34–36), which may influence postoperative dry eye outcomes. Therefore, effective management of postoperative dry eye holds significant clinical value. Our study aimed to investigate the therapeutic effect of 0.05% CsA eye drops in combination with DCBE eye gel for the treatment of DED after pterygium surgery.

DCBE eye gel, which is rich in various nutrients, promotes epithelial cell metabolism and regeneration, offering robust repair effects on the cornea and conjunctiva and helping to rapidly restore tear film stability (19). Its gel form also forms a protective layer on the ocular surface, reducing suture-induced corneal friction and effectively alleviating symptoms such as foreign body sensation (37). The efficacy of DCBE in improving tear film stability and relieving dry eye symptoms, particularly in patients with severe ocular surface damage or after pterygium surgery, has been demonstrated previously (38), with reported improvements in TFBUT (from 4.90 ± 2.26 s to 5.93 ± 1.72 s) and OSDI (from 42.55 ± 14.97 to 14.98 ± 7.58) after 4 weeks of treatment. However, in our study, the use of DCBE eye gel alone showed limited symptomatic relief, as reflected by no further OSDI score improvement after 1 month postoperatively. This limitation was overcome by incorporating 0.05% CsA eye drops. CsA eye drops are primarily utilized in ophthalmology to suppress ocular immune responses (39). Extensive clinical research has demonstrated that low-concentration CsA eye drops possess multiple therapeutic mechanisms in the treatment of DED (39, 40). Specifically, they can regulate the apoptosis of acinar cells, corneal epithelial cells, and conjunctival goblet cells, thereby increasing intracellular mucin storage (41, 42). Additionally, CsA effectively inhibits the activation and differentiation of T lymphocytes, leading to a reduction in the expression of conjunctival inflammatory markers, such as human leukocyte antigen, inflammatory factor interleukin-6 (IL-6), and matrix metalloproteinase-9 (MMP-9) (43). This inhibition exerts potent anti-inflammatory effects, alleviates dry eye symptoms, and reduces the risk of pterygium recurrence (44). Moreover, CsA can promote neurotransmitter release, thereby improving tear production feedback and increasing tear secretion (45). A previous study reported that after pterygium surgery, topical 0.05% CsA increased TFBUT by 5.9 s and SIT by 4.4 mm over 12 months (29). This beneficial effect was further validated by comparison with artificial tears, which only temporarily lubricate without resolving inflammation. A randomized controlled trial has demonstrated that 0.05% cyclosporine A significantly improves TFBUT after ocular surgery (13.56 ± 6.0 s vs. 9.50 ± 4.25 s, *p* = 0.004) (46). These findings align with the possible benefit of CsA in the postoperative setting.

In the assessment of therapeutic efficacy, our study adopts a comprehensive set of indicators, including OSDI, TFBUT, CFS, SIT, TMH, and ocular redness grading. This multi-faceted evaluation allows for a more holistic understanding of the treatment outcomes (47, 48). OSDI provides crucial insights into patients’ subjective experiences and the severity of their dry eye condition, while TFBUT effectively measures tear film stability (48). CFS accurately assesses corneal epithelial integrity, SIT quantifies tear production, TMH assesses tear secretion function, and ocular redness grading reflects the level of inflammation (49–52). Together, these indicators offer a detailed and reliable assessment of improvements in tear film stability, dry eye symptoms, and ocular surface health, highlighting the comprehensive impact of the treatment on patients’ quality of life and ocular health (53, 54). In our study, after drug treatment, all patients showed significant improvements in OSDI, TFBUT, CFS, SIT, and ocular redness grading compared to preoperatively (*p* < 0.05). Notably, the improvements in these parameters were more pronounced in the experimental group than in the control group. At 1, 2, and 3 months postoperatively, the experimental group exhibited significantly higher TFBUT and SIT values and lower OSDI and CFS scores compared to the control group (*p* < 0.05). This indicates that the combined drug application is more effective than monotherapy. This may be attributed to the ability of CsA to inhibit T-cell activation, thereby reducing inflammatory factors such as IL-6 and MMP-9, while DCBE eye gel might play a role in promoting corneal epithelial cell repair (19, 43). Together, they can improve tear film stability and the ocular surface microenvironment, achieving a therapeutic effect that monotherapy cannot match, and providing a more effective treatment option for DED following pterygium surgery. However, no significant differences in TMH and redness grading were observed between the two groups postoperatively (*p* > 0.05). This may be because most patients’ corneal epithelium had healed by 1 month postoperatively (12, 55), resulting in no significant changes in TMH. In terms of safety, the incidence of complications was 9.38% in the control group and 6.25% in the experimental group, with no significant difference between the two groups (*p* > 0.05). The complications observed in the control group included conjunctival congestion, blurred vision, and eye itching, whereas the experimental group experienced blurred vision and eye itching. The single case of conjunctival congestion in the control group may reflect a local inflammatory response to surgery or to the gel itself, as DCBE eye gel contains nutrients that could occasionally cause mild irritation. Blurred vision in both groups is an expected effect of gel-based formulations, which temporarily increase tear film viscosity and cause transient visual blurring, a phenomenon common with ophthalmic gels (56). Eye itching in both groups may be related to the preservative in the eye drops or to a mild hypersensitivity reaction to the active ingredients. Notably, patients experienced only mild irritation symptoms, which were alleviated after appropriate symptomatic treatment, and no systemic adverse reactions were observed, suggesting a favorable short-term safety profile of the drug application. Furthermore, the safety of DCBE has been reported in combination with other modalities (19) and as monotherapy for corneal disorders (37), while the epithelial protective effect of CsA has been demonstrated *in vitro* (41). During the 3-month follow-up period, no pterygium recurrence was observed in either group, indicating that the combination of pterygium excision and autologous limbal stem cell transplantation is effective in the short term. It should be noted that, as pterygium recurrence is typically defined as regrowth detected after at least 12 months of follow-up, longer observation is required to determine the true long-term recurrence rate.

The strengths of our study, which included combination therapy, comprehensive assessments, and acceptable sample size with well-balanced groups, allow our study results to provide meaningful evidence for short-term postoperative dry eye management. Nevertheless, our study has limitations. The follow-up period was relatively short. Future studies should extend the follow-up period to 6 months after drug withdrawal to comprehensively evaluate long-term efficacy and recurrence rates. Additionally, as a retrospective study, potential selection bias and reliance on medical records are inherent concerns that may affect the completeness of some assessments. Prospective studies with predefined protocols are needed to further validate our findings.

## 5 Conclusion

In conclusion, our study underscores the importance of evaluating DED in pterygium patients both preoperatively and postoperatively to better manage and prevent the development of postoperative DED. Moreover, our findings indicate that the combination of 0.05% CsA eye drops and DCBE eye gel is a promising approach for treating DED following pterygium surgery. This combined regimen appears to provide superior benefits compared to monotherapy.

## Data Availability

The original contributions presented in the study are included in the article/supplementary material, further inquiries can be directed to the corresponding author.
